# Gas-Phase Studies of NMR Shielding and Indirect Spin–Spin Coupling in ^13^C-Enriched Ethane and Ethylene

**DOI:** 10.3390/molecules29184460

**Published:** 2024-09-20

**Authors:** Marcin Wilczek, Karol Jackowski

**Affiliations:** Laboratory of NMR Spectroscopy, Faculty of Chemistry, University of Warsaw, Pasteura 1, 02-093 Warsaw, Poland; wilczek@chem.uw.edu.pl

**Keywords:** gas-phase NMR spectroscopy, ethane-^13^C_2_ and ethylene-^13^C_2_, nuclear magnetic shielding, indirect spin–spin coupling

## Abstract

^13^C and ^1^H NMR spectra were observed as the function of density in 1,2-^13^C-enriched ethane and ethylene for the pure gaseous compounds and their binary mixtures with xenon and carbon dioxide gases as the solvents. All the chemical shifts and indirect spin–spin couplings were linearly dependent on the solvent density. The appropriate NMR parameters (σ and ^n^J) in isolated ^13^C_2_H_6_ and ^13^C_2_H_4_ molecules and the coefficients responsible for the binary molecular interactions were determined and compared with previous similar measurements and selected calculated shielding data. The newly obtained ^13^C shielding values in the isolated ethane and ethylene molecules suggest visible secondary isotope effects due to the additional carbon-13 atom. All the investigated shielding parameters depend on intermolecular interactions, and the dependence of ^13^C shielding is much more marked. In contrast, the indirect spin–spin couplings in ^13^C_2_H_6_ and ^13^C_2_H_4_ molecules are almost independent of solvent molecules. Their ^n^J values determined in liquids over sixty years ago are generally consistent with the same ^n^J parameters in isolated ^13^C_2_H_6_ and ^13^C_2_H_4_ molecules.

## 1. Introduction

Ethane gas (C_2_H_6_) is a colorless, odorless, flammable, light-saturated hydrocarbon mostly available on a giant scale from natural gas. It can also be found dissolved in petroleum oil and is separated from the oil by fractional distillation. The carbonization of coal can obtain smaller quantities of ethane. The overwhelming mass of ethane is exploited to produce ethylene (ethene, C_2_H_4_) by steam cracking. Ethylene gas has similar physical properties to ethane but is much more reactive, as always occurs with unsaturated hydrocarbons. In the chemical industry, ethylene is used to produce polyethylene [1], the most popular plastic material in our everyday life. Moreover, the catalyzed hydration of ethylene gas over H_3_PO_4_ delivers technical ethyl alcohol (C_2_H_5_OH) [2] and the oxidation of ethylene gas leads to another important product, ethylene glycol (C_2_H_6_O_2_). Both the hydrocarbons—saturated ethane and unsaturated ethylene—are important industrial chemicals and have jointly almost the same final applications. For a similar reason, ethane and ethylene are the objects of our present experimental study in which the enriched carbon-13 molecules (^13^C_2_H_6_ and ^13^C_2_H_4_) are applied. We expect that the enriched molecules deliver more spectral information and permit us to obtain new insights into the electronic structure of ethane and ethylene.

Nuclear magnetic resonance (NMR) spectroscopy is an important method in analyses of organic molecules, particularly in the gas phase. Raynes, Buckingham, and Bernstein performed the first precise NMR study of gases in 1962 [3] exploring the proton spectra for selected gaseous compounds; ethane and ethylene were included in this research. The authors found that in a gas with low density, the nuclear magnetic shielding σ_A_(X) of X nucleus in A molecules can be written as an expansion in powers of the density ρ_A_:

σ_A_(X) = σ_0A_(X) + σ_1A_(X)ρ_A_ + σ_2A_(X)ρ_A_^2^ + … (1)

where σ_0A_(X) is the shielding for an isolated A molecule and σ_1A_(X) measures the intermolecular effect on nuclear shielding due to binary molecular collisions. The higher-order terms, starting from σ_2A_(X)ρ_A2_, are negligibly small for low-density samples. Then, the density dependence is linear and the two shielding parameters (σ_0A_ and σ_1A_) are readily available from Equation (1). Let us admit that the index X is added to the original RBB equation [3] because, at present, many types of magnetic nuclei can be observed by NMR methods. An additional index is required for the description of the applied multinuclear experiment.

For a binary mixture with a small amount of gas A, and gas B used as the solvent, the linear part of Equation (1) can be rewritten as follows:

σ_A_(X) = σ_0A_(X) + σ_1AA_(X)ρ_A_ + σ_1AB_(X)ρ_B_ + … (2)

where ρ_A_ and ρ_B_ are the densities of the A and B components, respectively, and σ_0A_(X) is the shielding at the zero-density limit, the same as in Equation (1). For a very low density of the A component, the σ_1AA_(X)ρ_A_ term is negligibly small, and after careful verification, it can be neglected. Finally, we have the following:

σ_A_(X) = σ_0A_(X) + σ_1AB_(X)ρ_B_ (3)

Both the experiments performed according to Equations (1) and (3) should deliver the same results for the shielding value in an isolated A molecule, σ_0A_(X). The precise description of σ_1AA_ and σ_1AB_ parameters is a bit more complex due to the binary collisions of A–A and A–B molecules. The parameters contain the change in shielding caused by the molecular collisions (σ_1(A-A)_ and σ_1(A-B)_) and the macroscopic effect of susceptibility change (σ_1b_) when the shielding measurements are performed using an external reference standard [3]. The σ_1b_ term can be estimated by knowing the investigated medium’s magnetic susceptibility, the NMR sample’s shape, and its orientation relative to the direction of the external magnetic field [4]. Finally, it should be remembered that all the shielding parameters in Equations (1)–(3) are temperature-dependent and that their experimental values are usually measured at the standard temperature of 300 K [5].

## 2. Results

### 2.1. NMR Spectra of ^13^C-Enriched Ethane and Ethylene

The ^1^H NMR spectra of ethane and ethylene containing a natural abundance of ^13^C nuclei (1.07 percent [6]) are easily affordable for direct observation because the proton magnetic moment is the largest among all stable nuclei, the protons in each investigated molecule are magnetically equivalent, and rare possible ^1^H–^13^C spin–spin couplings can only insignificantly modify proton signals. This is due to the low natural abundance of ^13^C isotopes usually present in the samples (1.07%), which is still sufficient for the fast observation of ^13^C spectra with modern NMR spectrometers. Spectrometers use the Fourier transform (FT) method and broadband decoupling from protons to detect carbon spectra. It is well illustrated by the ^1^H and ^13^C NMR spectra of ethane and ethylene as both spectral methods practically give strong singlet signals for C_2_H_6_ and C_2_H_4_ molecules [7,8]. Everything is changed when the ethane and ethylene molecules are fully enriched in the carbon-13 isotope [9,10]. The ^1^H and ^13^C NMR spectra of the ^13^C_2_H_6_ and ^13^C_2_H_4_ samples become more complex because neither two carbon-13 nuclei nor all protons are magnetically equivalent and their spectra have the forms A_3_A′_3_XX′ and AA′A″A‴XX′, respectively, where the A symbols stand for protons and X for carbon-13 nuclei. This means that the shielding values of ^1^H and ^13^C nuclei practically remain the same as for the samples with a natural abundance of ^13^C nuclei (assuming that the additional isotope effects from the second ^13^C nucleus in both the molecules are negligibly small [9]), but many new spin-spin couplings are observed and available for precise determination, as always occurs in such a case [11].

**Figure 1:**
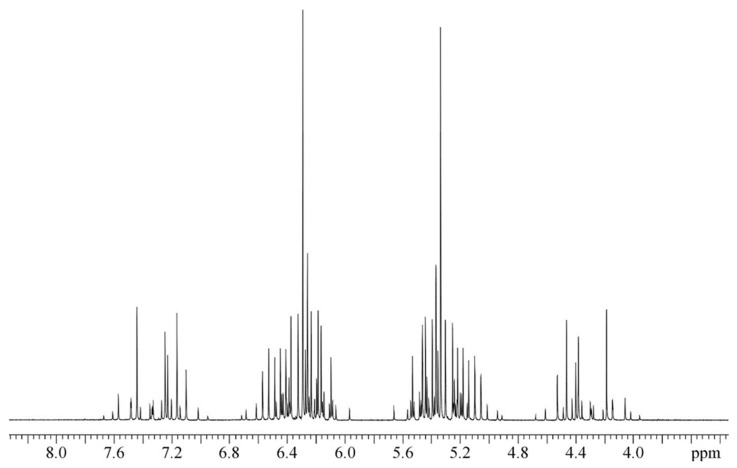
The 125 MHz ^13^C NMR spectrum of ethane gas doubly enriched in carbon-13 and without proton decoupling at the spectrometer field 11.7 T. The sample contains pure ^13^C_2_H_6_ at an approximate pressure of 40 bar. Pure liquid TMS is an external reference standard for carbon-13 chemical shifts.

**Figure 2:**
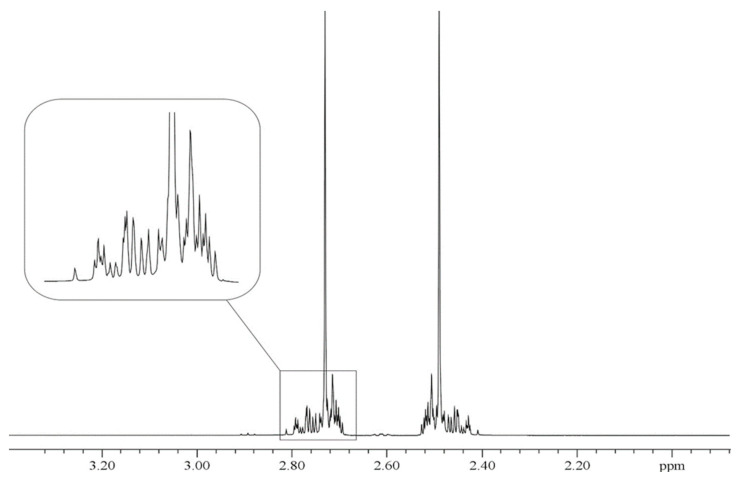
The 500 MHz ^1^H NMR spectrum (the A_3_A_3_′ fragment of the A_3_A′_3_XX′ spin system) of the same sample as in Figure 1. The enlarged part shows the complex structure of this spectrum due to all the possible spin-spin couplings between ^1^H and ^13^C nuclei. Its 50 NMR lines are partially overlapped. Liquid TMS is applied as an external reference standard of proton chemical shifts.

Figure 1 presents the ^13^C spectrum of gaseous doubly ^13^C-enriched ethane. The XX′ part of the A_3_A_3_′XX′ spin system is observed by ^13^C NMR, which consists of 67 separate NMR signals. All the spectral lines are well resolved, which helps with their precise analysis. In contrast, the ^1^H NMR spectrum presented in Figure 2 is less readable because many proton signals are overlapping. This results from the general rule that ^13^C spectra cover a much larger shielding range (approx. 250 ppm [12]) than the proton spectra with a shielding range of several ppm. It is also seen in the width of the present ^13^C spectrum of ^13^C_2_H_6_ (≈3.8 ppm in Figure 1) and only approx. 0.40 ppm in Figure 2 for ^1^H NMR measurement.

**Figure 3:**
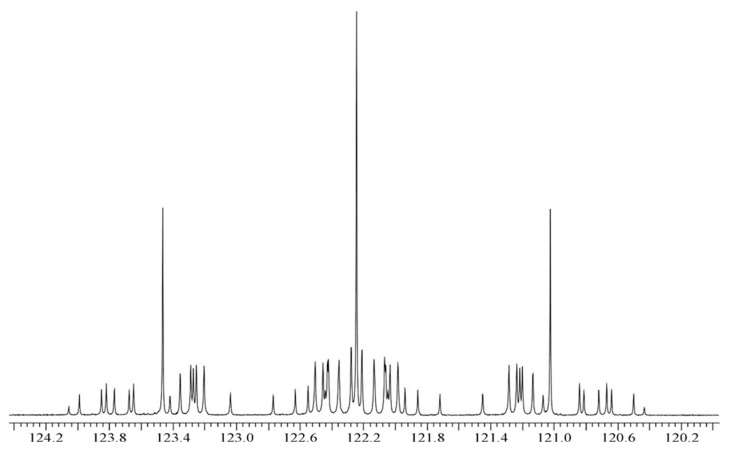
The 125 MHz ^13^C NMR spectrum (at 11.7 T) of ethylene enriched in carbon-13 and without decoupling from protons (the XX′ part of the AA′A″A‴XX′ spin system, with 144 partially overlapping NMR lines). The gas sample contains pure ^13^C_2_H_4_ gas with a pressure of approx. 45 bar. Liquid TMS is used for the measurements of carbon-13 chemical shifts.

**Figure 4:**
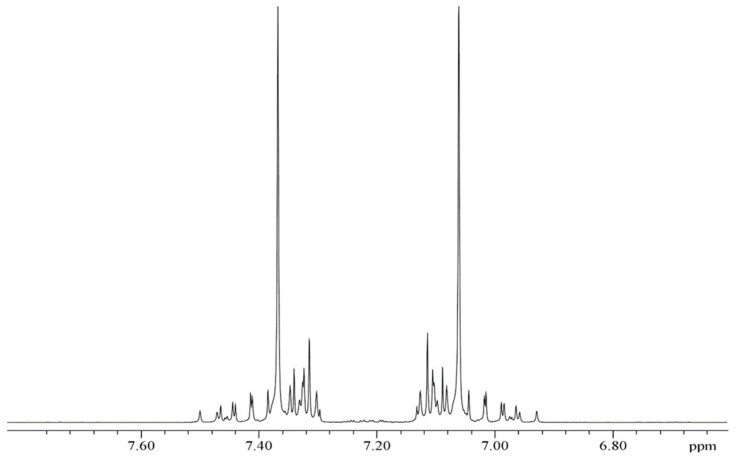
The 500 MHz ^1^H NMR spectrum of the same sample is shown in Figure 3. It is the AA′A″A‴ part of the AA′A″A‴XX′ spin system with 132 mostly overlapping signals. The proton chemical shifts are measured relative to external liquid TMS.

The spectra presented in Figure 1 and Figure 2 remain qualitatively unchanged when ethane-^13^C_2_ is mixed with some “inert” gases like xenon (Xe) or carbon dioxide (CO_2_); however, their spectral lines are slightly affected, as predicted by Equation (3). This is due to the binary collisions of the solute and solvent molecules in the gas phase. The ^13^C and ^1^H NMR spectra of doubly ^13^C-enriched ethylene presented in Figure 3 and Figure 4 show the same features as above described for ^13^C_2_H_6_ but are even more complex. It arises from the hindered rotation of the carbon-carbon double bond that makes all the protons in the ^13^C_2_H_4_ molecule magnetically nonequivalent. The increasing number of spin-spin couplings leads to more complicated ^13^C and ^1^H NMR spectra (AA′A″A‴XX′), as shown in Figure 3 and Figure 4.

### 2.2. Density-Dependent NMR Shielding in ^13^C_2_H_6_ and ^13^C_2_H_4_ Molecules

Figure 1 and Figure 2 reveal that the ^13^C and ^1^H spectra of ethane-^13^C_2_ are quite complex, as usual for the A_3_A′_3_XX′ spin system. It makes the above NMR spectra a little more difficult to interpret but delivers full information on all the spin–spin coupling constants with properly performed computer simulation. The same is true for the NMR spectra of ethylene-^13^C_2_ presented in Figure 3 and Figure 4, though the case of the AA′A″A‴XX′ spin system is even a little more complicated. However, all the spectra in Figure 1, Figure 2, Figure 3 and Figure 4 have an axis of symmetry and permit one to directly read all the chemical shifts and, consequently, the precise shielding parameters for all investigated samples.

**Figure 5:**
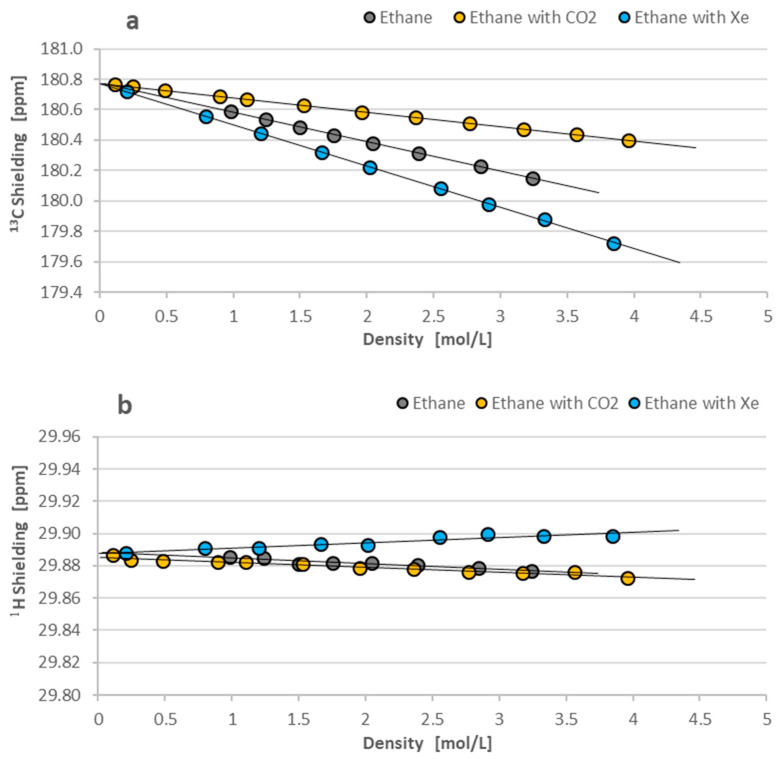
(**a**,**b**) Dependence of the ^13^C and ^1^H shielding on density observed in pure 1,2-^13^C-ethane and its binary mixtures with carbon dioxide (CO_2_) and xenon (Xe) as the gaseous solvents. The measurements were performed relative to liquid TMS accepting its shielding equal to 186.37 ppm for carbon-13 nuclei (**a**) and 32.815 ppm for protons (**b**) [13]. The error bars are marked by the size of experimental points. Let us note that the protons’ shielding scale is 5 times expanded compared to the ^13^C scale for better visibility.

The present ^13^C and ^1^H magnetic shielding measurements are established from NMR chemical shifts relating to external liquid TMS. Both the investigated nuclei are “light nuclei”, and the simplified form of Equation (4) is fairly satisfactory:

(4) $$\delta_{i}=\frac{\sigma_{TMS}-\sigma_{i}}{1-\sigma_{TMS}}\approx \sigma_{TMS}-\sigma_{i}$$

where σ_TMS_ and σ_i_ are the shielding values of TMS and investigated compounds, respectively. ^1^H and ^13^C magnetic shieldings were determined assuming shielding values for liquid TMS at 300 K: 32.815 ppm for protons and 186.37 ppm for carbon-13 nuclei [13]. Then, the obtained shielding parameters were plotted vs. the density of gases according to Equations (1) and (3), as illustrated by Figure 5a,b for ethane-^13^C_2_. The plots indicate the same intercept at the zero point density within an experimental error: approx. 180.775 ppm for ^13^C shielding and 29.889 ppm for ^1^H shielding. It confirms the equivalence of applications of the two methods in shielding readings: for pure solute compounds (according to Equation (1)) and observation in gaseous solvents (cf. Equation (3)). They have given us the parameters of shielding at the zero-density limit, which are equivalent to the results for the isolated ^13^C_2_H_6_ molecules. Moreover, the slopes recorded on the same figures have delivered the σ_1(A-A)_(X) and σ_1(A-B)_(X) coefficients, which show the intermolecular effects on shielding due to the binary molecular collisions. Figure 5a reveals the decrease in ^13^C shielding in ^13^C_2_H_6_ molecules with an increased gas density. It is a common effect for saturated hydrocarbons and all other chemical compounds without lone pairs of electrons in their molecules. The magnitude of σ_1(A-A)_(X) or σ_1(A-B)_(X) coefficient is the averaged effect of all possible bimolecular collisions in the gas phase. Figure 5a shows that the anisotropy of CO_2_ molecules can slightly diminish the σ_1_ intermolecular effect compared to the interactions observed in pure ethane molecules. In contrast, the isotropic xenon atoms containing more electrons diminish the ^13^C shielding in ^13^C_2_H_6_ due to the intermolecular effects.

Figure 5b presents the ^1^H shielding dependence on gas densities observed for 1,2-^13^C-ethane. In contrast to the ^13^C shielding from the previous picture, all the intermolecular effects observed on protons are negligible and remain within experimental errors (±0.01 ppm). Although the proton shielding scale in Figure 5b is five times enlarged relative to the ^13^C scale in Figure 5a, the effects of the binary molecular collisions are hardly seen for protons. In particular, in our opinion, the small increase in proton shielding with CO_2_ molecules is rather artificial and is within an experimental error [14,15]. Generally, it can be concluded that the density dependence of proton shielding in 1,2-^13^C-ethane has no diagnostic information on the intermolecular interactions observed in the gas phase. On the other hand, it proves that the electronic structure of ethane is very stable and fairly resistant to intermolecular interactions.

**Figure 6:**
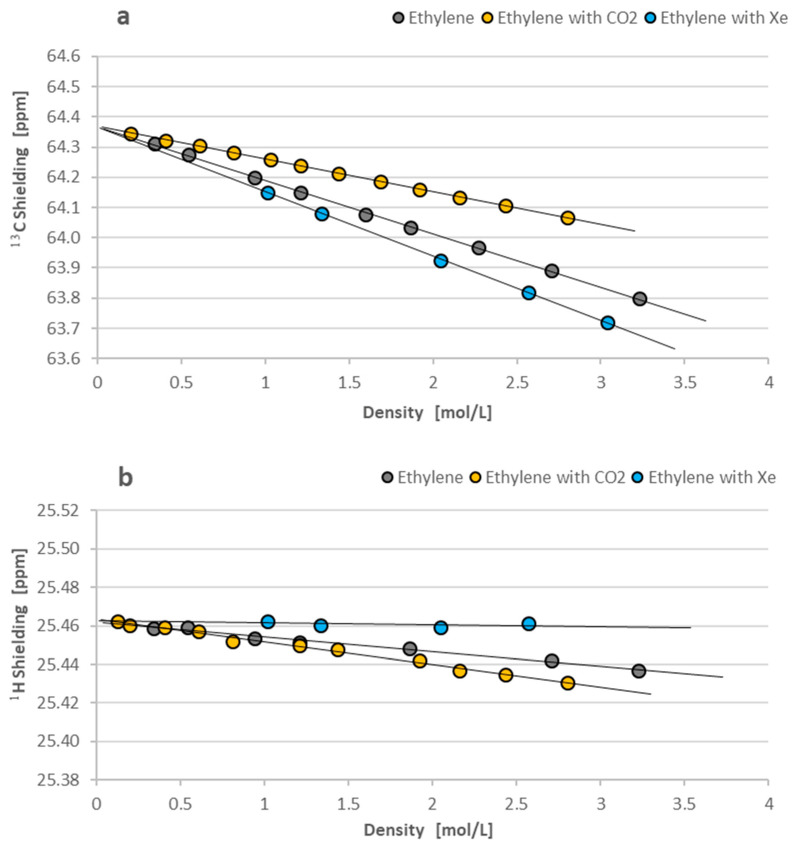
(**a**,**b**) Density-dependent ^13^C and ^1^H shielding of 1,2-^13^C-ethylene for the pure solute and its gaseous mixtures with CO_2_ and Xe. Liquid TMS (σ_C_ = 186.37 ppm, σ_H_ = 32.815 ppm) was applied as the external reference standard. The error bars are marked by the size of experimental points.

Below, we present in Figure 6a,b the shielding dependences on density for the ^13^C and ^1^H NMR measurements of 1,2-^13^C-ethylene in gaseous samples.

As seen in Figure 6a,b, the intermolecular interactions diminish the ^13^C and ^1^H magnetic shielding parameters in 1,2-^13^C-ethylene molecules in a very similar way to that for 1,2-^13^C-ethane: CO_2_ as the gas solvent provides the smallest decrease in ^13^C shielding, and Xe gas gives the largest negative effect. The magnitude of all the intermolecular effects in Figure 6a,b is similar to the previously observed changes in Figure 5a,b. The ^1^H shielding changes in 1,2-^13^C-ethylene are a bit more distinct (up to −0.03 ppm for CO_2_ solvent) but still small, as previously observed for 1,2-^13^C-ethane. Generally, no meaningful effects in the proton spectra can be detected for the intermolecular interactions in gaseous ^13^C_2_H_4_ (and ^13^C_2_H_6_) molecules with the selected gaseous solvents (CO_2_ or Xe). All the numerical data of our shielding measurements for 1,2-^13^C-ethane and 1,2-^13^C-ethylene are given in Table 1.

### 2.3. Isotropic Spin–Spin Coupling in ^13^C_2_H_6_ and ^13^C_2_H_4_ Molecules

Determining indirect spin–spin coupling constants requires the deconvolution of ^13^C NMR spectra, as described in the Experimental Section. Then, the observed parameters for all the spin–spin couplings (^n^J) can be analyzed similarly as was carried out for shielding using the linear part of density-dependent spin–spin couplings. The method has been previously used to observe ^1^J(^29^Si-^19^F) spin–spin coupling in gaseous SiF_4_ [16].

^n^J_A_(XY) = ^n^J_0A_(XY) + ^n^J_1AA_(XY)ρ_A_ (5)

^n^J_A_(XY) = ^n^J_0A_(XY) + ^n^J_1AB_(XY)ρ_B_ (6)

In the above equations, the XY symbols describe the indirect spin–spin coupling between X and Y nuclei across n chemical bonds. In our current presentation, the symbol A stands for the investigated compound (^13^C_2_H_6_ or ^13^C_2_H_4_) and B for a selected gaseous solvent (CO_2_ or Xe). Generally, the J-couplings in hydrocarbons are fairly stable and frequently remain almost unchanged due to intermolecular interactions in the gas phase [17], as happens for example in the methane molecule [18]. In contrast, heteroatoms in molecules can dramatically change the scale of ^1^J density dependence; such effects are observed for the ^1^J(CF) coupling in CD_3_F [19] or ^1^J(CH) in CH_2_F_2_ [20].

**Figure 7:**
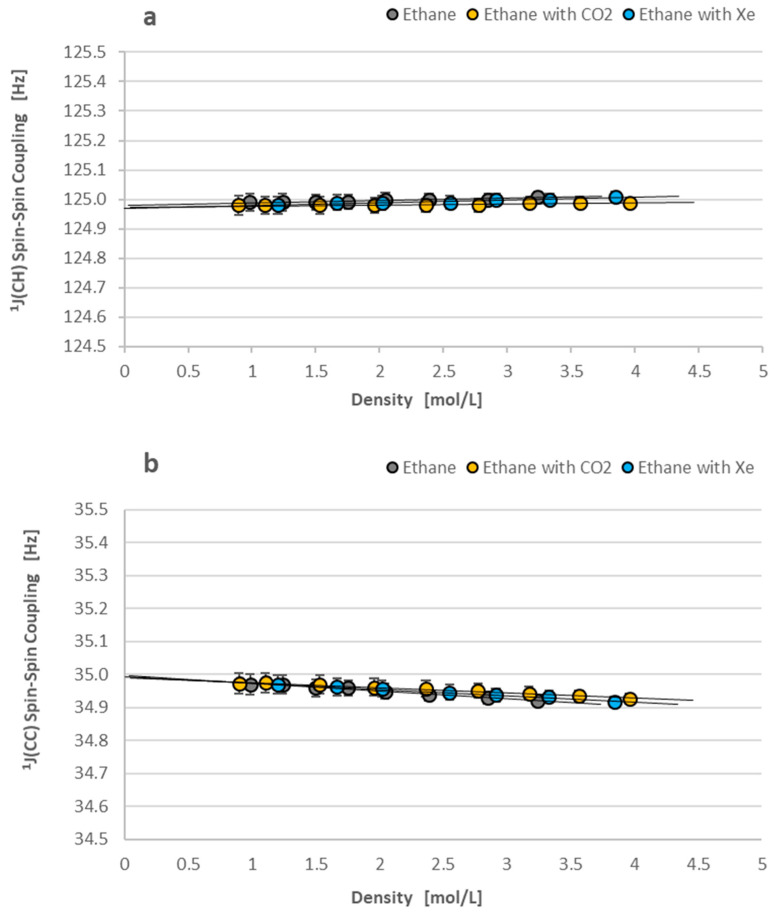
(**a**,**b**) The ^1^J(CH) and ^1^J(CC) isotropic spin–spin couplings in ^13^C_2_H_6_ as density functions at 300 K. Both plots show the negligible influence of intermolecular interactions on the one-bond couplings (^1^J(CH) in (**a**) and ^1^J(CC) in (**b**)) in ethane-^13^C_2_.

Four different spin–spin couplings are observed for the A_3_A_3_′XX′ spin system in ^13^C_2_H_6_ and six others for the AA′A″A‴XX′ spin system in ^13^C_2_H_4_. We have selected only the two most important couplings for the presentation in detail for each of the investigated hydrocarbons: ^1^J(CH)—because this coupling is available from NMR measurements performed for samples with the natural abundance of carbon-13 nuclei when the ^1^J(CC) is seen only in the enriched carbon-13 samples. The first case means the possibility of comparison with numerous other measurements, and the second case offers a new value for the parameter that is measured for the first time in the isolated molecules. Below, Figure 7a,b illustrates the linear density dependence of ^1^J(CH) and ^1^J(CC) spin–spin coupling in ethane-^13^C_2_ on gas density, as shown by Equations (5) and (6).

Figure 7a,b presents the typical value of ^1^J(CH) coupling (≈125 Hz) for the sp^3^ carbon hybridization of electrons in saturated hydrocarbons [12]. The ^1^J(CC) equal to 35 Hz is also typical for the one-bond ^13^C-^13^C coupling observed in saturated hydrocarbons [12]. It is more important that both the spin–spin couplings across one chemical bond in ethane are practically independent of intermolecular interactions. It proves ethane’s stable electronic structure, which remains unchanged to external molecular perturbations.

Finally, all the measurements performed on spin–spin coupling for the isolated ^13^C_2_H_6_ and ^13^C_2_H_4_ molecules are presented in Table 2, separately for each chemical compound. As shown in Table 2, all the J-couplings across more than one chemical bond are small and not differentiated by gas solvents as well; their graphical presentation was deliberately omitted in our presentation. In the next section, the new results on J-couplings are discussed and compared with similar earlier measurements and some data from quantum chemical calculations.

## 3. Discussion

### 3.1. Nuclear Magnetic Shielding

The current study provides the first measurements of ^13^C and ^1^H NMR shielding in ^13^CH_2_H_6_ and ^13^CH_2_H_4_ gases as the function of density according to Equations (1)–(3), presented in Figure 5 and Figure 6, and summarized in Table 1. As seen, the gas phase measurements allow for the precise determination of shielding parameters σ_0_(C) and σ_0_(H) free from intermolecular interactions and, therefore, equivalent for isolated molecules. Similar studies were performed earlier but using ethane and ethylene with the natural abundance of carbon-13 isotope. This means that the objects of previous shielding studies were slightly different: ^13^CH_3_^12^CH_3_ and ^13^CH_2_^12^CH_2_ for ^13^C NMR investigations, and mostly ^12^C_2_H_6_ and ^12^C_2_H_4_ for ^1^H experiments. The secondary isotope effect from the additional carbon-13 in the studied molecules is expected to be small. Therefore, we can safely compare all the shielding results on proton and carbon-13 in the isolated ethane and ethylene molecules. Table 3 gives the ^13^C and ^1^H shielding values from various experimental and theoretical investigations. The experimental data are limited to the σ_0_(C) and σ_0_(H) values and calibrated to a new scale of ^13^C shielding in which σ_0_(C) for an isolated CO molecule is equal to 0.6 ± 0.9 ppm [21]. The precision of ^1^H shielding measurements is much better and equal to ±0.005 ppm when liquid external TMS is applied as the reference standard [13]. The experimental σ_0_(C) and σ_0_(H) data shown in Table 3 are fairly consistent. It confirms that the secondary isotope effects in shielding are generally small. The calculated data of ^13^C and ^1^H shielding in ethane and ethylene molecules are inconsistent and mostly depend on the applied method of calculations.

As mentioned, Table 3 presents consistent σ_0_(C) and σ_0_(H) experimental data for ethane and ethylene. The consistency looks even better if the measurements of ^13^C shielding performed at higher temperatures [25] are excluded from our discussion for a while because it is of a bit lower precision. Such a measurement contains two unknown temperature contributions to shielding, one from the investigated molecule (ethane or ethylene) and the other from the reference molecule (methane in this case). The remaining three other σ_0_(C) results were obtained practically at the same temperature as for ethane and ethylene. Our present measurements give slightly larger ^13^C shielding values, 180.775 ppm for ^13^CH_2_H_6_ and 64.367 ppm for ^13^CH_2_H_4_ than the previous results [24,33]. It allows us to estimate the secondary isotope effects in carbon shielding: ^1^Δ(^13/12^C) ≈ +0.25 ppm for ethane and ^1^Δ(^13/12^C) ≈ +0.20 ppm for ethylene. The molecules with heavier isotopes have generally increased NMR shielding [34], supporting the above conclusion of ^1^Δ(^13/12^C) detection in carbon-13 doubly enriched ethane and ethylene.

Table 3 also contains the ^13^C and ^1^H shielding values for ethane and ethylene dissolved in liquid chloroform (CHCl_3_). The deshielding effects are observed in every case typical for aliphatic hydrocarbons [24]. The decrease in ^13^C shielding is more significant and correlates with the σ_1(A-B)_(C) parameters given in Table 1. The same is true for proton shielding in the investigated molecules but the decrease in ^1^H shielding is considerably smaller and limited only to a fraction of 1 ppm. Nevertheless, the intermolecular effects in shielding are significant in every condensed phase and require removal from the experimental results if the exact shielding values in molecules are needed. It is possible only to explore the measurements performed in the gas phase.

The results in Table 3 are given without error bars because the shielding scales of ^13^C and ^1^H nuclei have been defined earlier with the error bars as ±0.9 ppm and ±0.005 ppm, respectively. We have used the original suggestions citing other authors’ results and saved our data with the error bars in Table 1. The small error bars in Table 1 make sense because the current measurements are precisely performed relative to liquid TMS as the external reference standard. When the shielding scale of ^13^C is improved, one can easily obtain better values for ethane-^13^C_2_ and ethylene-^13^C_2_. Meanwhile, the comparison of various data in Table 3 illustrates only various experimental and theoretical investigations of shielding.

Ethane and ethylene are relatively small molecules, and the advanced ab initio methods based on the GIAO (Gauge Included Atomic Orbitals) approach [35,36], like CCSD (Coupled Cluster Singlets and Doubles) or CCSD(T) (Coupled Cluster Singlets and Doubles with Perturbative Triple Corrections) [37,38], can be applied for their shielding calculations [34]; such an approach to carbon-13 shielding is superior, as seen in Table 3. The modern state-of-the-art shielding calculations are very advanced, including all the intra- and intermolecular contributions, and the relativistic effects in shielding if required [39]. Various other more approximate methods can be used for shielding calculations in larger molecules: from HF (Hartree-Fock) to FCI (Full Configuration Interaction), MCSCF (Multi-Configuration Self-Consistent-Field), CC (Coupled Claster) approximation, and MP (Møller–Plesset) perturbation theory [40].

As shown, the σ_0A_ parameters of Equations (1)–(3) can be precisely determined and compared with previous NMR measurements and advanced quantum chemical calculations. The second-order terms of the same equations are much more complex and deliver many problems connected with the details of experimental studies performed in the gas phase. The σ_1_ parameters (also known as the second virial coefficient in Equations (1)–(3)) cannot be easily compared from various laboratories because the applied methods, or even different NMR spectrometers, may introduce significant discrepancies in the measurements of intermolecular effects. Precise quantum calculations of interacting large molecules like ethane or ethylene are still unavailable. The above problem certainly requires careful investigation in the future but exceeds the framework of the present work. For this reason, we have transferred the discussion on the σ_1_ parameters of ethane-^13^C_2_ and ethylene-^13^C_2_ to the Supplementary Materials.

### 3.2. Indirect Spin–Spin Coupling

**Figure 8:**
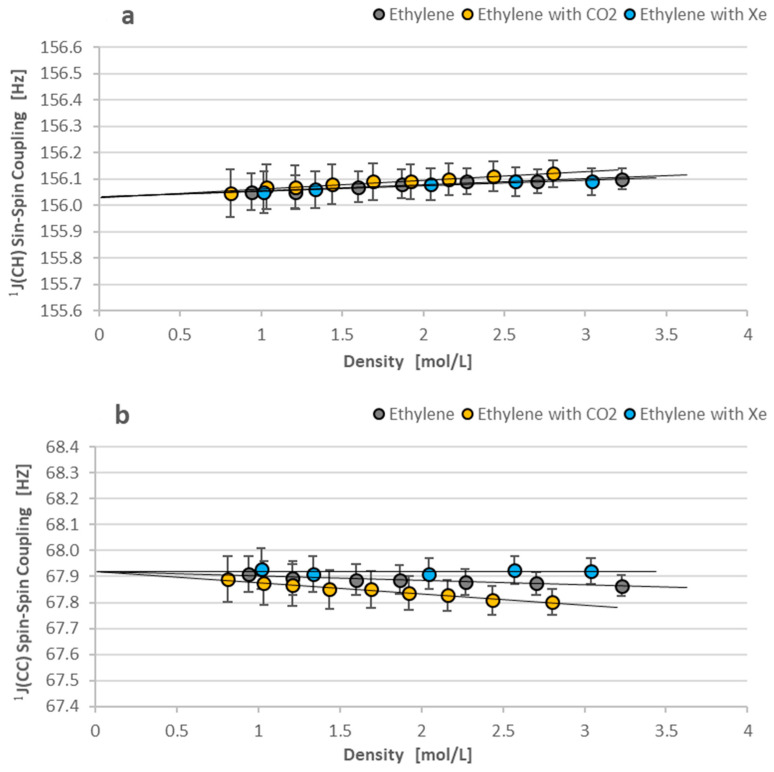
(**a**,**b**) The one-bond spin–spin couplings (^1^J(CH) in (**a**) and ^1^J(CC) in (**b**)) in ethylene-^13^C_2_ molecules. Their density dependence on gas density is still minimal but a little more differentiated by solvents than for ethane-^13^C_2_.

Figure 7 and Figure 8 present the minimal dependence of ^1^J(CC) and ^1^J(CH) spin–spin couplings on density in our study. The minimal effect can be better understood if we compare it with the ^1^H density-dependent shielding in Figure 5b and Figure 6b. Proton shielding is changed in the range of 0.02 ppm for ethane-^13^C_2_ and 0.03 ppm for ethylene-^13^C_2_. This means a change of approx. 10 and 15 Hz, respectively, in the ^1^H NMR spectrum (1 ppm ≈ 500 Hz). The ^1^J(CC) and ^1^JCH) spin–spin couplings vary only from 0.1 to 0.15 Hz in the same experimental conditions, i.e., two orders of magnitude less than ^1^H shielding.

Table 4 shows our recent results of spin–spin coupling data based on well-resolved ^13^C NMR spectra and extrapolated to the zero-density point. They are compared with the results of ^13^CH_2_H_6_ and ^13^CH_2_H_4_ obtained in condensed environments like liquid solutions in CCl_4_ [9], liquefied hydrocarbons at low temperatures [10], and liquid crystals [25]. Let us note that the change of samples has limited influence on the observed values of spin–spin coupling; quite different experiments show only minimal effects due to intermolecular interactions in this case. One more feature of the discussed data must be especially underlined. The first analyses of spin–spin coupling in ^13^CH_2_H_6_ and ^13^CH_2_H_4_ were performed over 60 years ago [9,10] using only available low-quality ^1^H NMR spectra. In our opinion, it is something fantastic and unusual in experimental NMR studies.

The comparison of plots in Figure 7b and Figure 8b reveals a more solvent-differentiated ^1^J(CC) coupling in ethylene-^13^C_2_ than the ^1^J(CC) in ethane-^13^C_2_. It is obviously due to a carbon-carbon double bond in the ethylene molecule. This effect is not confirmed in Table 4 if the results from various experiments are compared. However, the ^1^J(CC) of ethane and ethylene in Figure 7b and Figure 8b are similarly diminished due to intermolecular interactions. Table 4 confirms the decreased ^1^J(CC) values of ^13^CH_2_H_6_ and ^13^CH_2_H_4_ molecules in the condensed phases [9,10,25].

The results of the spin–spin couplings of ^13^CH_2_H_6_ and ^13^CH_2_H_4_ shown in detail in Table 2 are a bit better for isolated molecules and can be used as benchmarks in quantum chemical calculations. Table 4 also contains some selected calculated data of spin-spin couplings in ethane and ethylene molecules obtained from different computational methods [36,37,38,39,40,41,47]. The discrepancy between the experimental and theoretical results is well-marked. It arises from the difficulty in performing spin-spin coupling calculations utilizing quantum chemical methods [39,48]. In contrast to shielding calculations, more varied methods of calculations are applied and the different basis sets are focused on the inner electrons in the molecules. Approximate DFT (Density-Functional Theory) methods are more frequently used in studies of indirect J-coupling [49]. The above problems of such a comparison were recently discussed in detail when presenting the experimental and calculated ^n^J values for fluoromethanes [50].

## 4. Materials and Methods

### 4.1. Chemical Compounds and Samples Preparations

1,2-^13^C-ethane and 1,2-^13^C-ethylene (both with 99% enrichment in carbon-13 nuclei from Sigma-Aldrich, Poznań, Poland) plus carbon dioxide and xenon (99.99% Messer, also delivered by Sigma-Aldrich in lecture bottles) were used to prepare the samples without further purification. The gas samples were prepared via the condensation of gases from the calibrated part of a vacuum line into the 4.0 mm o.d. glass tubes (approx. 5.5 cm long), which were then sealed with a little torch. The volumes of the sample tubes and the vacuum line were measured using mercury. The studied gases (^13^C_2_H_6_ and ^13^C_2_H_4_) were observed as pure compounds in the range of approximately 7 to 50 bar or applied as solutes at low constant pressure (0.7 bar) and mixed with various quantities of the gaseous solvents (CO_2_ and Xe) up to a similar range of pressure (7–50 bar). The high-quality liquid solvents (Sigma-Aldrich) were carefully dehydrated and degassed in a vacuum line before the preparation of the liquid solutions with the enriched gaseous solutes. The gas samples were fitted into 5 mm o.d. standard NMR tubes (Wilmad 528-PP, Buena, CA, USA) with liquid cyclohexane-d_12_ in the annular space for the deuterium lock system. It also permitted us to gain additional control of the ^13^C and ^1^H chemical shifts because the shielding values of the liquid cyclohexane-d_12_ are precisely known (σ_liq_(^13^C) = 160.513 ppm and σ_liq_(^1^H) = 31.686 ppm) [51].

### 4.2. NMR Spectra

One-dimensional ^13^C and ^1^H NMR spectra were acquired on a Varian UNITYplus-500 FT spectrometer, Santa Clara, CA, USA (11.7 T) at the 125.88 and 500.62 MHz transmitter frequencies for the ^13^C and ^1^H nuclei, respectively. Ethane-^13^C_2_ was measured using a Varian sw5 (5 mm switchable_nmr_tm) probe, while ethylene-^13^C_2_ required the ID_PFG (indirect_nmr_tm) 5 mm probe. All the measurements were performed at the stabilized temperature of 300 K. The FID acquisition time (AQ) and scan numbers (NS) were variable with sample density due to the efficient spin-rotation relaxation and set up as follows: for ethane-^13^C_2_ (^1^H), AQ = 4–7s and NS = 16–256; for ethane-^13^C_2_ (^13^C), AQ = 3–6s and NS = 72–2000; for ethylene-^13^C_2_ (^1^H), AQ = 1.5–3s and NS = 500–2000; and for ethylene-^13^C_2_ (^13^C), AQ = 1.5–3s and NS = 1000–1600. D1 and DS parameters were not applied in this study. The spectral width was used from 400 to 800 Hz. The substitution method was applied to measure the ^13^C and ^1^H NMR chemical shifts relative to external liquid TMS [6]. The absolute magnetic shielding of TMS (32.815 ppm for protons and 186.37 ppm for ^13^C nuclei in a cylindrical tube parallel to the external magnetic field) [13] was used to convert the NMR chemical shifts into the absolute shielding values of ^13^C_2_H_6_ and ^13^C_2_H_4_. All the bulk susceptibility corrections (BSCs) that did not influence shielding in isolated molecules were removed from our experimental results in Figure 5 and Figure 6 and Table 1 and Table 3 using susceptibility data [52] and the appropriate formula for parallel orientation of cylindrical tubes relative to the external magnetic field [4]. More details on the second virial coefficients (σ_1_) and the BSC estimation are given in the Supplementary Materials.

### 4.3. Analysis of Spectral Parameters

Three to five spectra were recorded for each sample at various times to avoid accidental errors, and to increase the precision of the obtained results. It has allowed us to estimate the possible maximal errors in the NMR experiments. All the measurements were performed for a random order of solvent density. The total line-shape (TLS) program [53], as part of the PERCH Software (version 1/96) [47,54], was applied for the full deconvolution of the ^13^C NMR spectra of ethane-^13^C_2_ and ethylene-^13^C_2_. It permitted a more precise determination of all the spin coupling constants in the investigated compounds. Let us note that the appropriate proton spectra of investigated molecules were less resolved than the carbon spectra, as the ^1^H shielding scale is much smaller than that for ^13^C NMR. Finally, we analyzed the results for pure ^13^C_2_H_6_ and ^13^C_2_H_4_ using Equations (1) and (5) in the full density range. The gas mixtures required the application of Equations (3) and (6), and the measurements were performed for shielding and J-couplings in the limited density range, as shown in Figure 7a,b, and Figure 8a,b. This means the molar ratio (x_M_) ^13^C_2_H_6_ changes from 0.040 to 0.0092 in CO_2_ and from 0.036 to 0.0094 in Xe. Similar data were obtained for ^13^C_2_H_4_, as follows: 0.009–0.0029 for CO_2_ as the solvent and 0.011–0.0038 for Xe. Larger x_M_ values were assigned for lower densities of the solvent gases.

## 5. Conclusions

The present study yields experimental results for the ^13^C and ^1^H magnetic shielding and spin–spin coupling parameters of 1,2-^13^C-enriched ethane and ethylene in the gas phase. For the first time, the ^13^C and ^1^H NMR spectra of the investigated compounds were observed for pure compounds and their binary mixtures with xenon and carbon dioxide gases in a wide range of densities. All the shielding and spin–spin couplings were linearly dependent on gas density. The appropriate NMR parameters (σ_0_ and ^n^J_0_) in isolated ^13^C_2_H_6_ and ^13^C_2_H_4_ molecules and the coefficients responsible for the binary molecular interactions were determined and compared with similar previous measurements and selected calculated data based on modern quantum chemical methods. The comparison of intermolecular effects in shielding is less reliable because many experimental details influence the final value of σ_1_ parameters. We have extended the presentation of intermolecular shielding effects in the Supplementary Materials, adding some previous results obtained for ethane and ethylene with natural abundance of carbon-13 [3,21,47,55,56,57].

The newly-obtained ^13^C shielding values in the isolated ethane-^13^C_2_ and ethylene-^13^C_2_ molecules suggest visible secondary isotope effects (^1^Δ(^13/12^C)) due to the second carbon-13 atom in the studied molecules. The present study confirms that all the shielding parameters in ^13^C_2_H_6_ and ^13^C_2_H_4_ are dependent on intermolecular interactions and the dependence of ^13^C shielding is much more marked. Generally, the decrease in shielding is observed in a more dense molecular isotropic environment. In contrast, the indirect spin–spin couplings in ^13^C_2_H_6_ and ^13^C_2_H_4_ molecules are almost independent of solvent molecules. Their ^n^J values determined in liquids over sixty years ago are consistent with the same ^n^J parameters in isolated ^13^C_2_H_6_ and ^13^C_2_H_4_ molecules. However, the current measurements reveal that the ^1^J(CC) coupling in ethylene is more sensitive to molecular interactions than the appropriate ^1^J(CC) in ethane. The present study confirms that all the parameters of shielding and spin–spin coupling in ethane and ethylene molecules are more or less sensitive to intermolecular interactions, and this fact cannot be neglected if precise NMR measurements are required.

Small molecules like ^13^C_2_H_6_ and ^13^C_2_H_4_ are especially interesting for the development of new quantum-chemical methods of calculations. The theoretical techniques are always more or less approximate tools, and their improvement is crucial for chemical applications. With new experimental data, the various new methods of quantum calculations can be easily verified, especially when the experimental data are available for small isolated molecules. Our present research delivers experimental data on proton and carbon-13 shielding, numerous J-coupling constants, and isotopic effects in shielding for ^13^C_2_H_6_ and ^13^C_2_H_4_ molecules.

## Figures and Tables

**Table 1:** Nuclear magnetic shielding of gaseous 1,2-^13^C-enriched ethane and ethylene measured at a temperature of 300 K from density dependence of the pure compounds and their binary mixtures with CO_2_ and Xe.

		Measured with Gaseous Solvent (B)	
Parameter of A Molecule	Pure A Gas	CO_2_	Xe
*NMR shielding in ^13^C_2_H_6_*			
σ_0_(C) [ppm]	180.775(2)	180.773(2)	180.776(3)
σ_1(A-B)_ (C) [ppm mL mol^−1^]	−193(3) *	−95(3)	−273(4)
σ_0_(H) [ppm] σ_1(A-B)_ (H) [ppm mL mol^−1^]	29.889(2) −4(3) *	29.885(2) −3(3)	29.889(2) 3(4)
*NMR shielding in ^13^C_2_H_4_*			
σ_0_(C) [ppm] σ_1(A-B)_ (C) [ppm mL mol^−1^]	64.367(3) −176(3) *	64.368(2) −108(3)	62.364(5) −213(3)
σ_0_(H) [ppm] σ_1(A-B)_ (H) [ppm mL mol^−1^]	25.462(2) −8(3) *	25.464(2) −12(3)	25.462(3) −1(3)

* σ_1(A-A)_ value for the pure solute gas.

**Table 2:** Spin–spin couplings of gaseous 1,2-^13^C-enriched ethane and ethylene are measured at 300 K from their density dependences and pure compounds and in binary mixtures with CO_2_ and Xe.

		Measured in Gaseous Solvent (B)	
Parameter of A Molecule	For Pure A Gas	CO_2_	Xe
*Spin–spin coupling in ^13^C_2_H_6_*			
^1^J_0_(CH) [Hz]	124.98(3)	124.97(3)	124.97(3)
^1^J_AB_(CH) [Hz mL mol^−1^]	9(4) *	4(4)	9(4)
^1^J_0_(CC) [Hz]	35.00(2)	34.99	35.00
^1^J_AB_(CC) [Hz mL mol^−1^]	−24(4) *	−16(4)	−20(4)
^2^J_0_(CH) [Hz]	−4.79(2)	−4.80(2)	−4.80(2)
^2^J_AB_(CH) [Hz mL mol^−1^]	7(4) *	5(3)	9(3)
^3^J_0_(HH) [Hz]	8.08(2)	8.09(2)	8.09(2)
^3^J_AB_(HH) [Hz mL mol^−1^]	−4(4) *	−5(3)	−6(3)
*Spin–spin coupling in ^13^C_2_H_4_*			
^1^J_0_(CH) [Hz]	156.03(2)	156.03(2)	156.04(3)
^1^J_AB_(CH) [Hz mL mol^−1^]	22(9) *	32(9)	19(12)
^1^J_0_(CC) [Hz]	67.92(2)	67.92(2)	67.92(3)
^1^J_AB_(CC) [Hz mL mol^−1^]	−17(7) *	−43(10)	1(14)
^2^J_0_(CH) [Hz]	−2.55	−2.54(2)	−2.55(2)
^2^J_AB_(CH) [Hz mL mol^−1^]	11(4) *	9(11)	9(10)
^2^J_0_(HH) [Hz]	2.53(2)	2.53(2)	2.54(4)
^2^J_AB_(HH) [Hz mL mol^−1^]	−39(8) *	−31(10)	−36(17)
^3^J_0_(HH-cis) [Hz]	11.81(2)	11.81(2)	11.81(3)
^3^J_AB_(HH-cis) [Hz mL mol^−1^]	−4(7) *	4(11)	−6(11)
^3^J_0_(HH-trans) [Hz]	19.18(2)	19.19(2)	19.18(3)
^3^J_AB_(HH-trans) [Hz mL mol^−1^]	−20(8) *	−11(10)	−17(12)

* J_AA_ value for the pure solute gas, cf. Equation (4).

**Table 3:** Experimental ^a^ and calculated ^b^ shielding parameters of ethane and ethylene molecules.

^13^C Shielding [ppm]		^1^H Shielding [ppm]	
exp. σ_0_(^13^C)	calc. σ_0_(^13^C)	exp. σ_0_(^1^H)	calc. σ_0_(^1^H)
*NMR Shielding in ^13^C_2_H_6_*			
180.775 ^c^	181.9 ^d^	29.889 ^c^	30.84 ^e^
180.55 ^f^	184.7 ^g^	29.894 ^h^	30.92 ^g^
180.5 ^i^	185.1 ^j^	29.87 ^k^	31.77 ^l^
181.04 ^m^	182.1 ^n^	29.97 ^m^	31.05 ^n^
(176.628) ^o^	185.5 ^p^	(29.526) ^o^	30.66 ^p^
*NMR Shielding in ^13^C_2_H_4_*			
64.367 ^c^	66.5 ^d^	25.462 ^c^	26.06 ^e^
64.24 ^f^	73.6 ^g^	25.468 ^h^	26.18 ^g^
64.1 ^i^	60.8 ^j^	25.43 ^k^	26.69 ^l^
64.55 ^m^	58.0 ^n^	25.47 ^m^	26.08 ^n^
(60.410) ^o^	69.4 ^p^	(24.979) ^o^	25.93 ^p^

^a^ Gas-phase results extrapolated to zero density and corrected to the absolute scale of ^13^C shielding (σ_0_ = 0.6 ± 0.9 ppm for ^13^CO molecule [21]), otherwise as described; ^b^ GIAO calculations as described; ^c^ this work; ^d^ CCSD(T) including vibrational corrections [22]; ^e^ MP2 corrected for rovibrational contributions [23]; ^f^ originally measured relative to liquid benzene [24] and converted to shielding as described in ref. [13]; ^g^ RAS-II calculations [25]; ^h^ measured relative to an isolated methane molecule [26] assuming CH_4_ proton shielding, σ_0_ = 30.633 ppm [27]; ^i^ measured in low-density gas [28] and corrected according to the actual ^13^C shielding scale [21]; ^j^ ref. [28]; ^k^ low-density gas [29]; ^l^ SCF [30]; ^m^ measured relative to an isolated methane molecule at 348K [26] and accepting carbon-13 σ_0_ (^13^CH_4_) = 195.01 ppm [31]; ^n^ SCF [21]; ^o^ in liquid CHCl_3_, present work; ^p^ from DFT studies [32].

**Table 4:** Experimental and ab initio calculated spin–spin coupling parameters of ^13^C-enriched ethane and ethylene molecules.

	Indirect Spin–Spin Coupling [Hz]	
Parameter	Experimental	Calculated
*Spin–spin coupling in ^13^C_2_H_6_*		
^1^J(^13^C,^1^H)	124.98 ^a^, 124.9 ^b^, 125.3 ^c^, 125.190 ^d^,	119.8 ^e^, 119.97 ^f^, 127.5 ^g^, 130.06 ^h^,
^1^J(^13^C,^13^C)	35.00 ^a^, 34.6 ^b^, 34.4 ^c^, 34.558 ^d^,	38.8 ^e^, 35.20 ^f^, 23.6 ^g^, 38.34 ^h^, 32.6 ^i^
^2^J(^13^C,^1^H)	−4.79 ^a^, −4.5 ^c^, −4.655 ^d^,	−5.3 ^e^, −5.05 ^f^, −4.3 ^g^, −5.33 ^h^
^3^J(^1^H,^1^H)	8.08 ^a^, 8.0 ^c^, 8.002 ^d^,	7.2 ^e^, 4.03 and 14.88 ^f^, 5.16 ^h^
*Spin–spin coupling in ^13^C_2_H_4_*		
^1^J(^13^C,^1^H)	156.03 ^a^, 156.3 ^b^, 156.4 ^c^, 156.302 ^d^,	147.7 ^e^, 150.21 ^f^, 155.3 ^g^, 154.2 ^j^, 163.4 ^k^
^1^J(^13^C,^13^C)	67.92 ^a^, 67.6 ^b^, 67.6 ^c^, 67.54 ^d^,	70.2 ^e^, 67.53 ^f^, 70.6 ^g^, 70.5 ^i^, 70.1 ^j^, 71.1 ^k^
^2^J(^13^C,^1^H)	−2.55 ^a^, −2.4 ^b^, −2.4 ^c^, −2.408 ^d^,	−3.3 ^e^, −3.58 ^f^, −1.0 ^g^, −4.1 ^h^, −1.3 ^k^
^2^J(^1^H,^1^H)	2.53 ^a^, 2.5 ^b^, 2.5 ^c^, 2.23 ^d^,	0.9 ^e^, 0.98 ^f^, 3.4 ^g^, 3.2 ^j^, 0.2 ^k^
^3^J(^1^H,^1^H*_cis_*)	11.81 ^a^, 11.7 ^b^, 11.7 ^c^, 11.62 ^d^,	10.4 ^e^, 11.79 ^f^, 11.7 ^g^, 11.0 ^j^, 12.8 ^k^
^3^J(^1^H,^1^H*_trans_*)	19.18 ^a^, 19.0 ^b^, 19.1 ^c^, 19.02 ^d^,	17.0 ^e^, 18.02 ^f^, 18.3 ^g^, 17.7 ^j^, 19.7 ^k^

^a n^J_0_ from the present ^13^C NMR experiments; ^b^ from ^1^H NMR spectra of ^13^C-enriched hydrocarbons in CCl_4_ solutions [9]; ^c 1^H NMR spectra of liquid ^13^C-enriched hydrocarbons at −70 °C [10]; ^d^ a selected example of ^13^C-enriched hydrocarbons dissolved in liquid crystals (LC), ^1^H and ^13^C NMR experiments [25]; ^e^ GIAO MCSCF calculations with more examples in ref. [25]; ^f^ CCSD [41], the two results for ^3^J(^1^H,^1^H) in ethane are for the two opposite orientations of H atoms in the stable structure of the ^13^CH_2_H_6_ molecule without averaging; ^g^ B97-3 [42]; ^h^ BHandH [43]; ^i^ B3LYP [44]; ^j^ B3LYP [45]; ^k^ SOPPA(CCSD) [46].

## Data Availability

Data are contained within the article and Supplementary Materials.

## Supplementary Materials

The following supporting information can be downloaded at: https://www.mdpi.com/article/10.3390/molecules29184460/s1, Table S1: The second virial coefficient of shielding for gaseous ethane and ethylene.
