# Supplementary material for: Gas-Phase Studies of NMR Shielding and Indirect Spin–Spin Coupling in 13C-Enriched Ethane and Ethylene
Source: Molecules. 2024 Sep 20;29(18):4460. doi: 10.3390/molecules29184460 (PMC11434548; doi:10.3390/molecules29184460)
Supplement: Supplementary file 1 [file molecules-29-04460-s001.zip › molecules-3106268-supplementary.pdf]

molecules

MDPI

Supplementary Materials

Gas-Phase Studies of NMR Shielding and Indirect Spin–Spin Coupling in ^{13}C-Enriched Ethane and Ethylene

Marcin Wilczek and Karol Jackowski*

Laboratory of NMR Spectroscopy, Faculty of Chemistry, University of Warsaw, Pasteura 1, 02-093 Warsaw, Poland; wilczek@chem.uw.edu.pl
*Correspondence: kjack@chem.uw.edu.pl

Supplementary Materials

Influence of intermolecular interactions on NMR shielding in gaseous ethane and ethylene

Eqs. 1-3 show that the first virial coefficients of shielding (σ_0, in isolated molecules) are independent of all the possible intermolecular interactions. In contrast, the second virial coefficients (σ_1) are due to the binary collisions of molecules if the density dependence of shielding remains linear [3]. In such a case, σ_1 contains the change in shielding caused by intermolecular interactions ($\sigma_{1(A\text{-}A)}$) and the bulk susceptibility correction (BSC, σ_{1b}). The precise determination of σ_{1b} is difficult because numerous factors can slightly change its value. Generally, it is assumed that σ_{1b} = - (2/3)χ_v for a long cylindrical NMR tube if the external magnetic field B_0 is perpendicular (⊥) to the tube and σ_{1b} = + (4/3)χ_v if the similar tube is parallel (∥) to B_0. χ_v is the diamagnetic volume susceptibility of a sample. Everything looks easy, but it is rather complex when NMR experiments are performed in different laboratories at various times. The following details can influence the estimated values of BSC:

1. The formulas σ_{1b} = - (2/3)χ_v, and σ_{1b} = + (4/3)χ_v are valid only for infinite long tubes ideally oriented relative to B_0, and the requirements are never exactly true in experiments.
2. Old NMR spectrometers with electromagnets used the perpendicular (⊥) B_0 orientation, while modern machines used the parallel system (∥) inside superconducting magnets.
3. Different laboratories used variable gas samples, with neither the length nor the diameter of samples being the same.
4. The values of volume susceptibilities (χ_v) changed over time, and their slightly different values were applied to original papers.
5. Last but not least, the precision of the gas sample preparation is also an important factor that can influence the estimation of σ_{1b}.

Below, in Table S1, we collected the available experimental σ_1 results obtained for ethane and ethylene in the gas-phase measurements. The results are presented in the c.g.s. units [ppm mL mol^{-1}] because all previous experiments were published this way.

Table S1. The second virial coefficient of shielding for gaseous ethane and ethylene.

Sample orientation[a]	Value of σ_{1b} [ppm mL mol^{-1}] C_2H_6/C_2H_4	Ethane		Ethylene	
		σ_{1A}	$\sigma_{1(A\text{-}A)}$	σ_{1A}	$\sigma_{1(A\text{-}A)}$
		^{1}H NMR [ppm mL mol^{-1}]			
⊥	-57.3/-40.8	-74(8)[b]	-17(8)[b]	-48(2)[c]	-7(2)[c]
		-73(2)[d]	-16(2)[d]	-51(2)[e]	-10(2)[e]
‖	114.6/81.7	108(3)[f]	-6(3)[f]	64(4)[f]	-18(4)[f]
		105(3)[g]	-9(3)[g]	70(3)[g]	-12(3)[g]
		111(2)[h]	-4(2)[h]	74(2)[h]	-8(3)[h]
		^{13}C NMR [ppm mL mol^{-1}]			
⊥	-57.3/-40.8	-281(16)[i]	-224(16)[i]	-241(35)[i]	-200(35)[i]
‖	114.6/81.7	-85(3)[g]	-200(6)[g]	-81(6)[g]	-163(6)[g]
		-78(2)[h]	-193(3)[h]	-94(4)[h]	-176(4)[h]

[a] Relative to an external magnetic field (B_0); [b] Raynes et all. [3]; [c] Gordon and Dailey [55]; [d] Rummens [56]; [e] Trappeniers and Oldenziel [57]; [f] Smith and Raynes [4]; [g] Bennet and Raynes [26]; [h] present results; [i] Jackowski and Raynes [24].

As seen in Table S1, the general agreement between various measurements is preserved, but discrepancies between different data are also seen. Unfortunately, the quantum chemical calculations of $\sigma_{1(A\text{-}A)}$ for ethane and ethylene are still unavailable. Such calculations are probably possible but require lots of work and computer time. Let us hope that the calculations will be available soon and that they confirm the NMR experiments in the gas phase for ethane and ethylene.
